# Neutrophils in Gliomas

**DOI:** 10.3389/fimmu.2017.01349

**Published:** 2017-10-26

**Authors:** Matteo Massara, Pasquale Persico, Ornella Bonavita, Valeria Mollica Poeta, Massimo Locati, Matteo Simonelli, Raffaella Bonecchi

**Affiliations:** ^1^Humanitas Clinical and Research Center, Rozzano, Italy; ^2^Department of Medical Biotechnologies and Translational Medicine, Università degli Studi di Milano, Milan, Italy; ^3^Humanitas University, Rozzano, Italy

**Keywords:** neutrophils, glioma, inflammation and cancer, immunotherapy, cancer immunotherapy, chemokines

## Abstract

Neutrophils are the most abundant white blood cells and are the first recruited to inflammatory sites. Neutrophils are an important component of the tumor stroma and can exert both anti-tumoral and pro-tumoral activities, depending on their maturation and activation state. In human gliomas, the number of circulating and infiltrating neutrophils correlates with the severity of the disease, indicating a prognostic and possible pro-tumoral role for these leukocytes. In glioma preclinical models, neutrophils promote tumor growth and orchestrate the resistance to anti-angiogenic therapies. Nevertheless, recent data indicate that neutrophils can be activated to directly kill tumor cells or to orchestrate the anti-tumoral response. Here, we review current knowledge about the role of neutrophils in glioma and their possible involvement in new strategies to improve current cancer therapies.

## Introduction

Gliomas represent approximately 80% of malignant brain tumors with an estimated annual incidence of 6.6 per 100,000 individuals in the USA. Before publication of the revised World Health Organization (WHO) Classification of Tumors of the central nervous system (CNS) in 2016, gliomas were historically classified into four major histological groups (grades I–IV), according to their microscopic characteristics (such as cytological atypia, anaplasia, mitotic activity, microvascular proliferation, and necrosis) and clinical behavior. These tumors were also divided into astrocytomas (WHO grade I–IV), oligodendrogliomas (WHO grade II–III), and mixed oligoastrocytomas (WHO grade II–III) depending on their putative cells of origin.

Grade IV glioma or glioblastoma (GBM) is the most common and lethal primary brain tumor in adults and notoriously has a highly aggressive clinical course with a median survival time ranging from 12.2 to 18.2 months and less than 5% of patients alive after 5-year from initial diagnosis. In contrast, gliomas of lower grade (WHO grade II–III), accounting for approximately one-third of all gliomas, are usually less aggressive tumors with a highly variable clinical course that is not adequately predicted on the basis of their histologic class. Some are indolent tumors, but others quickly progress to a more aggressive form or to GBM over years. Genome-wide molecular-profiling studies have now revealed a comprehensive mutational landscape for all major types of human gliomas occurring in adults and in children (1). In the revised 2016 WHO Classification of Tumors of the CNS, the advances in our molecular understanding of gliomas are integrated in a novel, multilayered approach to disease categorization that incorporates both histological and molecular information to better define many tumor entities and their prognosis.

Despite all these molecular efforts, the current standard of care for newly diagnosed GBM has remained unchanged over the past 10 years and includes maximal safe resection followed by radiotherapy plus concomitant and adjuvant chemotherapy with temozolomide (2). Current feasible therapeutic options for lower-grade gliomas vary with the extent of resection, histologic class, grade, and the results of molecular testing (IDH mutations and 1p and 19q co-deletion) and include clinical monitoring, radiation alone, sequential, or concomitant chemo-radiation. Outcome for patients with recurrent/progressive disease is dismal, and no therapeutic intervention has been associated with durable survival benefit. According to that, considerable interest has been directed in the development of more effective therapies including innovative therapeutic approaches.

However, clinical trials evaluating intensifying cytotoxic therapy, targeting dysregulated cell signaling pathways (3), and blocking angiogenesis (4) failed to prolong patients’ survival. Giving the exciting and durable clinical benefits achieved for a number of other challenging cancers, recent efforts have been directed toward the modulation of the immune system by various therapeutic interventions also in the neuro-oncology field. Until now immunotherapeutic strategies for gliomas were only partially successful, further underlying the strong immunosuppressive potential of these tumors (5). This article aims to review the role of neutrophils in glioma, their role in the immunosuppression, and resistance to therapies and possible strategies to harness their anti-tumoral potential to fight gliomas.

## The Immune Response in the CNS

The CNS has traditionally been recognized as an immune-privileged site lacking the potential for immunosurveillance due to the presence of the blood–brain barrier (BBB) and the absence of a conventional lymphatic system. Lack of immune response is provided by preclinical data demonstrating the prolonged survival of tissues grafted into brain that were otherwise rejected rapidly by the immune system when grown outside the CNS. However, this paradigm has been recently challenged and it is becoming increasingly clear that the CNS interacts dynamically with the systemic immune system. Recent data indicate that leukocytes can traffic to the CNS, even in the presence of an intact BBB, and that CNS antigens can reach peripheral lymph nodes by draining cerebrospinal fluid (CSF) through Virchow-Robin spaces (6, 7). Furthermore, in 2015, the presence of lymphatic vessels within the dural sinuses capable of shuttling leukocytes between CSF and deep cervical lymphatic vessels has been reported (8).

In homeostatic conditions, leukocytes are only present in the CSF and in the vessels and they do not enter in the brain parenchima. The only immune cells that are present in the CNS are parenchymal macrophages (microglia) that have a prenatal origin, are long-lived, and have no turnover with blood monocytes (9). Furthermore, in the brain, there are nonparenchymal macrophages named perivascular, meningeal, and choroid plexus macrophages that are strategically located at the CNS barrier and are thought to modulate leukocyte traffic and to sense blood danger signals (9).

Naive T cells do not readily enter the normal or inflamed CNS. Rather, T cells need to be highly activated to enter the brain, suggesting that they need to be primed in the periphery to be able to enter to this site (10). Inflammatory monocytes infiltrate the brain during autoimmune and neurodegenerative diseases with an intact BBB, indicating that they are actively recruited to the CNS. The CCR2/CCL2 axis seems to be crucial for the trafficking of monocytes across the BBB (11). Neutrophils can infiltrate the brain with a LFA-1-dependent mechanism during Alzheimer disease (12), and in a murine model of sepsis, neutrophils are actively recruited to the brain by the chemokines, such as CXCL1, CXCL2, and CXCL3 (13). In has to be noted that in humans the chemokines involved in brain recruitment of neutrophils can be different because mice do not express CXCL8 the major human neutrophil attractant.

## The Role of the Immune System in Glioma

By interfering with different aspects of tumor growth and responsiveness to therapy, the interplay between tumor cells and the immune system is emerging as a key modulator of tumor biology and one of the major determinants of cancer pathogenesis and progression (14). Increasing evidence indicates that tumors develop and progress by co-opting seemingly normal host cell types that reside in or are recruited to tumor microenvironment.

**Figure 1:**
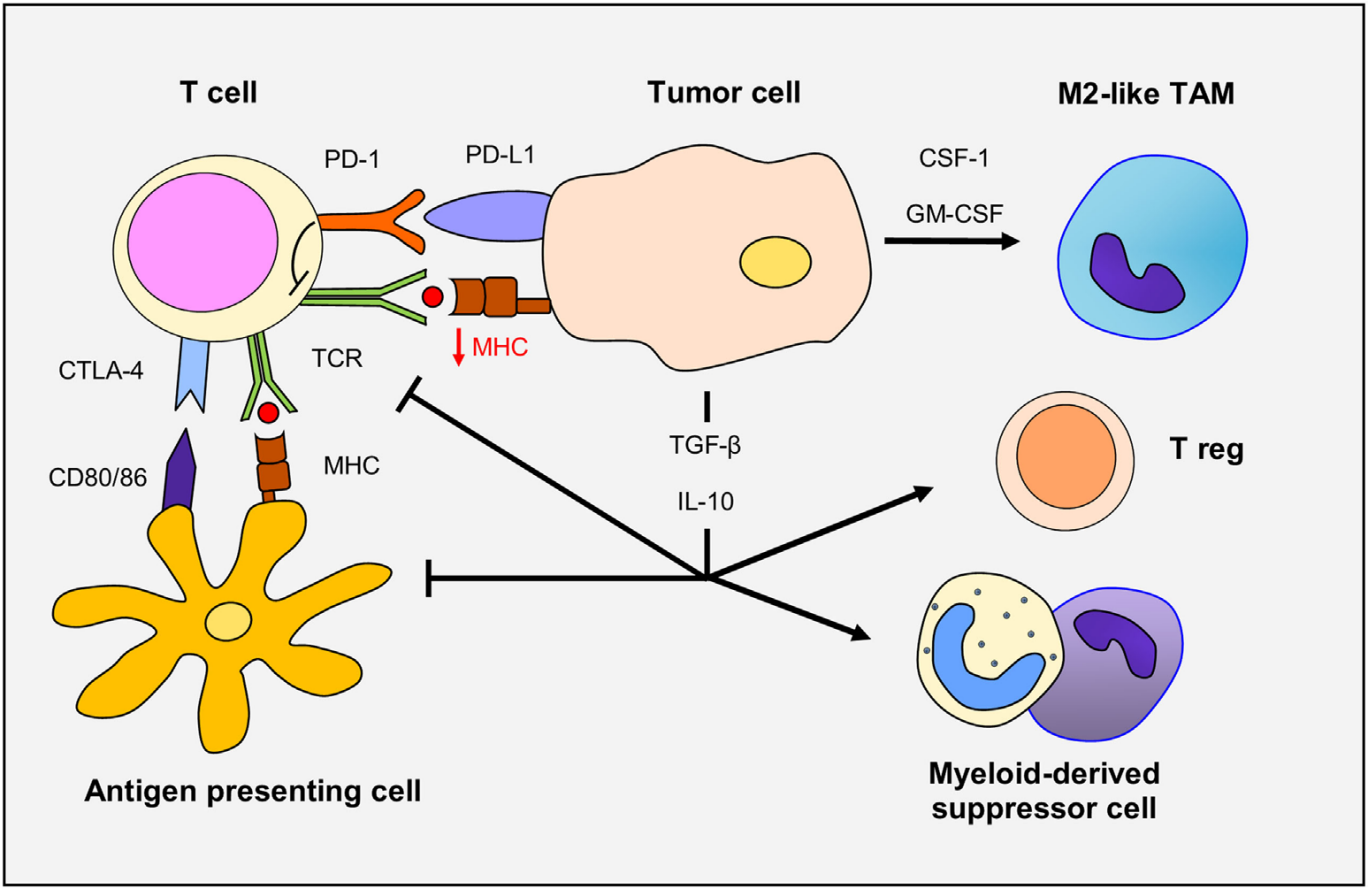
The immune response in gliomas. Glioma cells overexpress TGF-β and IL-10, immunosuppressive cytokines that decrease T-cell responsiveness and antigen-presenting cell function. Glioma cells down-regulate MHC-I and upregulate programmed death ligand-1 (PD-L1) expression leading to defective T-cell responsiveness. TCR activity is also dampened through CTLA-4 expression. TGF-β and IL-10 promote the recruitment and differentiation of regulatory T cells. Finally, secretion of CSF-1 and GM-CSF by glioma cells increase macrophage recruitment and polarization to M2-like phenotype.

In glioma patients, there is a profound and generalized immunosuppression, particularly within the context of cell-mediated immunity (Figure 1). The systemic immunosuppression is driven by overexpression by the glioma cells of soluble factors, such as prostaglandin E2, TGF-β, indoleamine 2,3-dioxygenase, and IL-10, all of which lead to decrease T-cell responsiveness to pro-inflammatory signals and ineffective presentation of tumor antigens by antigen-presenting cells (APCs). On the other side, the same mediators drive the recruitment of immunosuppressive cells, such as regulatory T cells and myeloid suppressor cells, to the tumor microenvironment. Moreover, corticosteroids (often used in glioma patients to diminish cerebral edema) are immunosuppressive, and temozolomide chemotherapy and radiotherapy can trigger prolonged lymphopenia. Several immunosuppressive alterations do occur within the glioma microenvironment. Glioma cells can down-regulate their own MHC-I complexes, thus becoming “invisible” to the adaptive immune system, as well as increase expression of immune checkpoint regulators, such as PD-1 (15–17). Specific blockade of PD-1 in combination with radiotherapy induced tumor regression and promoted long-term survival in animal models of glioma (18). The combination therapy of anti-CTLA-4 and anti-PD-1 has resulted in a 75% long-term remission in an orthotopic, immunocompetent murine GBM model. In this model, there were increased numbers of activated CD8^+^ and NK cells with reductions in suppressive immune cells in the tumor microenvironment (19). Additionally, combined blockade of CTLA-4, PD-1 and indoleamine dioxygenase, and anti-CTLA-4 therapy alone have resulted in long-term survival in other murine models of GBM and combined blockade of CTLA-4 and IL-12 was shown to increase numbers of effector T cells and decrease Tregs (20–22).

Multiple strategies to overcome immunosuppression and boost anti-tumor immune responses are now under evaluation in clinical trials, including vaccination with tumor-specific or tumor-associated peptides (or with administration of autologous APCs pulsed with such peptides), immunomodulatory monoclonal antibodies that target T-cell inhibitory signaling pathways to enhance physiological anti-tumor responses and adoptive T-cell transfer, which involves *ex vivo* clonal expansion or genetic engineering of T cells (23).

In addition to strategies aimed to T-cell activation, efforts were also done to target tumor-associated macrophages to inhibit glioma progression. Indeed, macrophages appear to be the main infiltrating immune cells in gliomas. Several approaches have been performed using inhibitors of the CSF-1 receptor (CD115). This treatment blocks maturation of monocytes and polarization of macrophages toward an M2-like pro-tumoral phenotype and resulted in increased survival of the GBM-bearing mice (24, 25). Other myeloid cells are present in the glioma microenvironment, including neutrophils (26). These leukocytes, until a few years ago, were considered as innocent bystanders in the tumor stroma, and their role in the tumor context was not investigated. On the contrary, emerging evidence indicates that neutrophils can play an important role in tumors having both pro-tumoral and anti-tumoral functions.

## Neutrophils and Cancer

Neutrophils are the most abundant leukocyte population in the bloodstream, and they are essential effectors of the inflammatory response and defense against pathogens. Even if neutrophils are mostly considered for their anti-microbial functions, it is now evident that they exert a broader role in the immune response (27).

In the cancer setting, neutrophils were considered for a long time only for their pro-tumoral activities because in many cancer types, neutrophilia and an elevated ratio of peripheral neutrophils-to-lymphocytes counts (NLR) are associated with poor prognosis (28, 29). They were considered the main cell type mediating immunosuppression and tumor promotion. Only recently their anti-tumoral potential has been reconsidered for several reasons. First, clinical studies have indicated that neutrophil infiltration is associated with a favorable prognosis in gastric cancer (30), in colorectal cancer (31), and in early stages of non-small-cell lung carcinoma (32). Second, preclinical data have indicated that circulating and tumor-infiltrating neutrophils are not a homogeneous population as previously considered. Tumor-derived cytokines induce the appearance in the blood of immature neutrophils with immunosuppressive activities (33) and neutrophils with an “aged” phenotype that are experienced cells with increased ability to respond to inflammatory stimuli that can have an anti-tumoral role (34). Also, in the tumor tissue, as macrophages, neutrophils are present in different polarized states: N1 anti-tumoral phenotype induced by IFN-β stimulation and N2 pro-tumoral phenotype induced by TGF-β and G-CSF stimulation (35). In addition, several preclinical data have indicated that neutrophils can be activated to kill tumor cells. Neutrophils mediate the therapeutic effect of monoclonal antibodies such as rituximab and trastuzumab in breast cancer (36) and lymphoma (37, 38) through antibody-dependent cellular cytotoxicity (ADCC). Neutrophils are the effector cells in the regression of Fas ligand (FasL)-expressing tumors through the expression of Fas (39). They also mediate the anti-tumor activity of the PKC modulator ingenol-3-angelate (PEP005) in skin tumors. PEP005 induces the recruitment and the activation of neutrophils that kill the tumor cells by ADCC (40). Neutrophils are again the effector cells in *Bacillus* Calmette–Guérin-based bladder tumor immunotherapy inducing death of cancer cells by release of TRAIL (41). Interestingly, reactive oxygen species production by neutrophils mediates the immune-mediated abscopal effect of radiotherapy that is tumor regression outside the radiation field (42). Finally, neutrophils are mediating the anti-tumoral activity of the tyrosine kinase inhibitor Cabozantinib in prostate cancer models. Cabozantinib induces the production of neutrophil chemotactic factors such as CXCL12 and HMGB1 that promote a strong infiltration and activation of neutrophils that kill tumor cells (43).

## Neutrophils and Glioma

As for other cancer patients, most of the glioma patients have a strong neutrophilia (44) due to overproduction by tumor cells of G-CSF, the growth factor for neutrophils (45, 46). G-CSF diverts BM hematopoiesis away from the lymphocyte lineage toward the granulocyte one and for this reason patients have a high NLR. NLR higher than 4 has been associated with poor prognosis when measured before treatments (47, 48), after the second surgery (49), and after temozolomide chemotherapy and radiotherapy (50). NLR less than 4 is associated to better prognosis but only in GBM expressing the wild-type gene IDH1, one of the genes more frequently mutated in malignant gliomas (51). Activation of neutrophils, measured by increased surface expression of CD11b, is an early predictor of tumor progression in GBM patients (52). Circulating neutrophils promote tumor growth inducing immunosuppression by production of arginase I (53). Interestingly, baseline neutrophil count in blood predicts bevacizumab efficacy in GBM patients. However, neutrophils are not involved in the anti-tumor response but are, on the contrary, the target of the anti-VEGF-A therapy being the main producers of this proangiogenic cytokine (54).

Neutrophils are also present within the tumor lesions (55), and there is a correlation between NLR and the degree of neutrophil infiltration (56). The number of infiltrating neutrophils is correlated with glioma grade and with acquired resistance to anti-VEGF therapy in GBM (55, 57). Clinical data suggest that neutrophils have a negative prognostic value in GBM patients, but the mechanism of their recruitment and their role in tumor growth are ill defined. In addition, preclinical studies have an important limitation due to differences in relative abundance of neutrophils in the circulation between human and mice (50–70 and 10–25%, respectively).

Neutrophils are recruited at tumor site by CXCL8 produced by FasL triggering on glioma cells (58, 59). Immunosuppressive neutrophils are also recruited by the chemotactic agent MIF produced by glioma cancer stem cells (60). At the tumor site, neutrophils secrete elastase that can aid glioma infiltration (61) and can directly induce the proliferation of GBM-initiating cells by producing S100A4 (57). Neutrophils are also contributing to the resistance to anti-angiogenic therapy (62). Consistently with their pro-tumoral role, depletion of neutrophils using the monoclonal antibody against Ly6G prolonged the survival of mice with developing gliomas (63). Moreover, mutant IDH1 glioma tumors that have a longer survival compared to wild-type IDH1 have a reduced neutrophil infiltration (64).

## Conclusion and Future Perspectives

**Figure 2:**
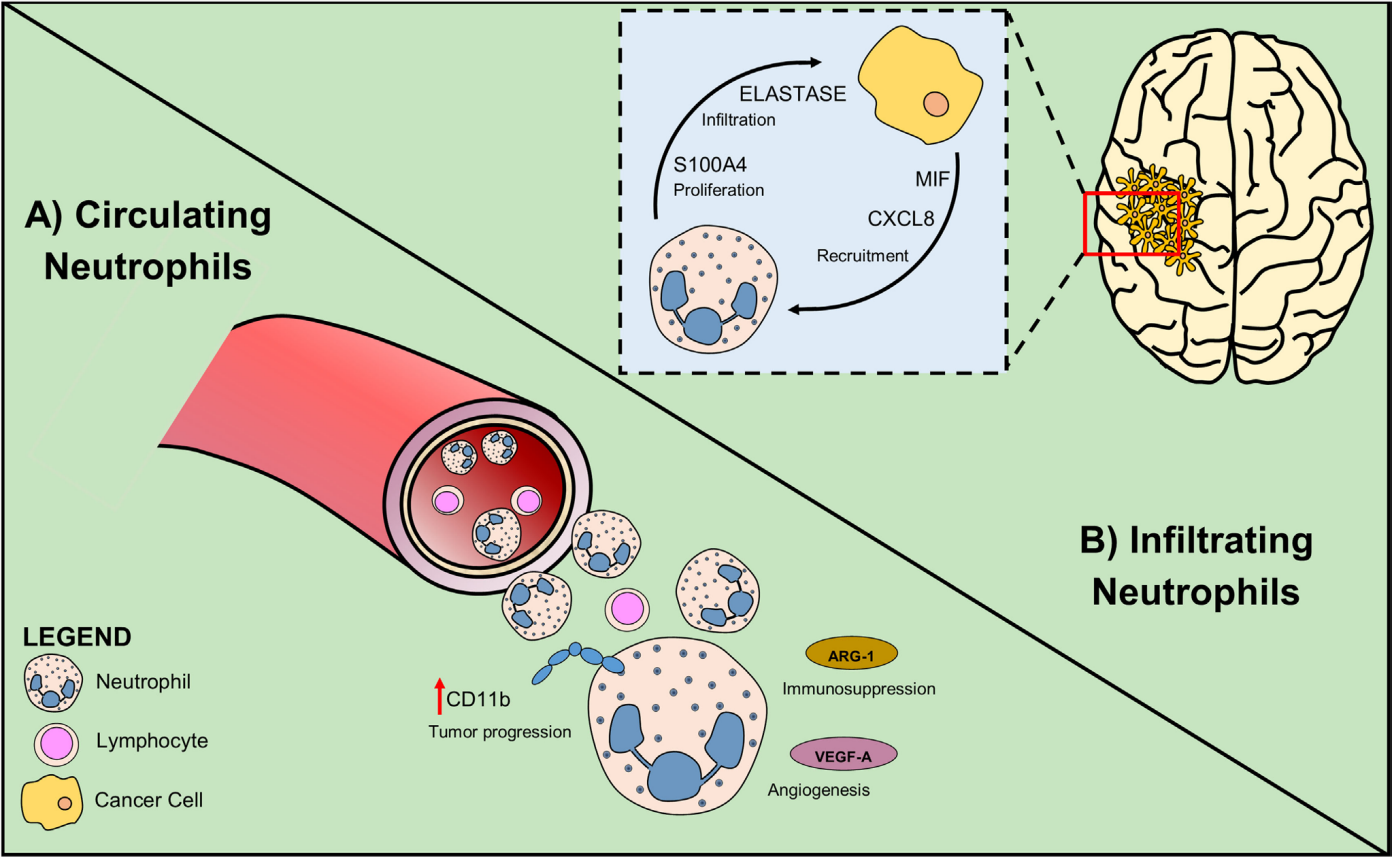
Circulating and infiltrating neutrophils sustain glioma growth. **(A)** In glioma patients, high NLR is associated with poor prognosis. Neutrophil activation measured as increased CD11b level, is an early sign of tumor progression. Secretion of Arginase-1 by neutrophil promotes immunosuppression and VEGF-A production induces angiogenesis. **(B)** Glioma cells attract neutrophils by producing the chemoattractants CXCL8 and MIF. Tumor-associated neutrophils exert their pro-tumoral role expressing elastase that sustains glioma infiltration and S100A4 that increases tumor proliferation rate.

To date, most studies performed on glioma patients suggest that neutrophilia and neutrophil infiltration at tumor site have a negative prognostic role in the survival and response to anti-angiogenic therapies (Figure 2). Therefore, most of the approaches are targeted at inhibiting neutrophil migration to the tumor site. Because in other tumors, neutrophils can directly exert an important antineoplastic activity and improve the efficiency of current therapies, such as anti-cancer antibodies and checkpoint inhibitors, it will be important to uncover this possibility also in glioma.

Consideration should be given to the possibility to improve monoclonal antibody therapies by combination with treatments that promote the differentiation of neutrophils into an anti-tumor phenotype with higher ADCC activity. Interestingly, two treatments, type I IFNs (65) and TGF-β targeting (56), that can promote neutrophil differentiation toward an anti-tumoral phenotype are inducing glioma regression. Current therapies may also be combined with treatments that promote selective recruitment of anti-tumoral neutrophils at the tumor site (66). Finally, the ability of neutrophils to migrate in the inflamed brain can be exploited to deliver anti-cancer drugs in gliomas after surgery, reducing regrowth of tumors (67).

## Author Contributions

All the authors have contributed to this review by writing and critically evaluating the literature.

## Conflict of Interest Statement

The authors declare that the research was conducted in the absence of any commercial or financial relationships that could be construed as a potential conflict of interest.
